# Metabolomic and Transcriptomic Changes Induced by Potassium Deficiency During *Sarocladium oryzae* Infection Reveal Insights into Rice Sheath Rot Disease Resistance

**DOI:** 10.1186/s12284-021-00524-6

**Published:** 2021-09-17

**Authors:** Jianglin Zhang, Zhifeng Lu, Tao Ren, Rihuan Cong, Jianwei Lu, Xiaokun Li

**Affiliations:** 1 Key Laboratory of Arable Land Conservation (Middle and Lower Reaches of Yangtze River), Ministry of Agriculture and Rural Affairs, People’s Republic of China, Wuhan, 430070 China; 2 Microelement Research Center, Huazhong Agricultural University, Wuhan, 430070 China

**Keywords:** Lipid peroxidation, K deficiency, *Sarocladium oryzae* infection, Sheath rot, Metabolome and transcriptome

## Abstract

Rice sheath rot disease caused by *Sarocladium oryzae (S. oryzae)* infection is an emerging disease, and infection can cause yield losses of 20–85%. Adequate potassium (K) application is a feasible strategy for rice tolerance to *S. oryzae* infection. However, little is known about the metabolic mechanisms regulated by K that allow rice to cope better with *S. oryzae* infection. The present study performed a comparative metabolome and transcriptome analysis of rice with different K nutrition statuses before and upon *S. oryzae* infection. *Sarocladium oryzae* infection triggered a hydrogen peroxide (H_2_O_2_) burst, and K starvation aggravated the accumulation of H_2_O_2_ in the flag leaf sheath (FLS), which resulted in lipid peroxidation. Likewise, K deficiency altered the lipid homeostasis of the host plants by hyperaccumulation of 1-alkyl-2-acylglycerophosphoethanolamine. K starvation decreased the content of glycoglycerolipids including monogalactosyldiacyglycerol and digalactosyldoacylglycerol during *S. oryzae* infection, which destroyed the stability of bilayer membranes. In contrast, sufficient K supply increased antioxidant-related transcript expression (for example, the genes related to glutathione-S-transferase biosynthesis were upregulated), which activated the antioxidant systems. Additionally, upon *S. oryzae* infection, K starvation amplified the negative impacts of *S. oryzae* infection on flag leaf photosynthetic potential. These results provide new insight into the role of K in alleviating *S. oryzae* infection. Adequate K supply decreased the negative impacts of sheath rot disease on rice growth by alleviating lipid peroxidation and maintaining lipid homeostasis.

**Supplementary Information:**

The online version contains supplementary material available at 10.1186/s12284-021-00524-6.

## Background

Rice (*Oryza sativa* L.) is a widely grown crop worldwide, that plays an important role in food security. Sheath rot (ShR) disease caused by *Sarocladium oryzae* ((Sawada) W. Gams & D. Hawksw) is an emerging threat to rice production, and the infection of which causes yield losses of 20–85% (Sakthivel 2001; Bigirimana et al. 2015). *S. oryzae* generally invades rice via the flag leaf sheaths (FLSs) during the booting stage, and the typical symptoms are grayish-brown lesions occurring on the FLSs that gradually evolve into rotting of the entire FLSs (Hittalmani et al. 2016). Our previous study demonstrated that rotting of the FLSs inhibited the exportation of non-structural carbohydrates from the stems and FLSs to the grains during the grain-filling process, which profoundly decreased the seed-setting rate and final grain yield (Zhang et al. 2019a). Therefore, the suppression of rotting is crucial for enhancing rice tolerance to *S. oryzae* infection.

The tolerance capacity of host plants is often associated with cell-tissue metabolic processes, such as amino acid and lipid metabolism, which directly take part in plant-pathogen interactions (Fagard et al. 2014; Rojas et al. 2014). Lipids provide the basic component of cell membranes, which act as the first line of defense against pathogen invasion (Adigun et al. 2021). To attack plants, fungal pathogens generally secrete toxins that target plant lipid metabolism and trigger an oxidative burst. A previous study reported that infection by *S. oryzae* results in the secretion of cerulenin, which may disturb fatty acid biosynthesis and lipid metabolism (Bigirimana et al. 2015). As an immune response, an oxidative burst protects plants against pathogenic infection; however, the consistent accumulation of hydrogen peroxide (H_2_O_2_) induces lipid peroxidation, which, when it exceeds a certain threshold level, may contribute to membrane damage and cell death (Triantaphylidès et al. 2008; Nita and Grzybowski 2016). The tolerance that plants usually display against infection by a fungal pathogen occurs by maintaining the stability of cellular membranes and regulating lipid homeostasis (Raffaele et al. 2009). Examples include regulating the contents of monogalactosyldiacylglycerol (MGDG) and digalactosyldiacylglycerol (DGDG), which are essential for maintaining the integrity and stability of cellular membranes in response to abiotic and biotic stresses (Perlikowski et al. 2016; Zhang et al. 2019b). *S. oryzae* infection may also induce oxidative stress, further resulting in cell death (Bigirimana et al. 2015). However, the alterations in the lipid metabolic processes of the host plant that cope with *S. oryzae* infection remain unknown. Considering that ShR encloses young panicles, it is difficult to control this disease by using chemical fungicides. Thus, regulating the host plant’s metabolism to improve its tolerance ability is an important strategy against *S. oryzae* infection.

Potassium (K) is the most abundant of the univalent cations in rice, and its application profoundly improves plant tolerance to infection by most fungal pathogens (Amtmann et al. 2008). Despite the fact that K is not metabolized in the host plant, the activation of enzymes, maintenance of cell membrane stability, and promotion of antioxidant biosynthesis are modulated by cytoplasmic K^+^ concentrations, and all these processes are essential for plant-pathogen interactions (Marschner 2012). Notable, plant K deficiency generally induces higher NADPH oxidase activity, which accelerates the accumulation of H_2_O_2_ in K-starved plants (Shin and Schachtman 2004). As mentioned previously, excessive H_2_O_2_ accumulation induces electrolyte leakage and causes cell death (Demidchik et al. 2014). Therefore, long-term K deficiency in rice may aggravate oxidative stress under biotic stress conditions, which manifests in K-starved plants as hyperaccumulation of H_2_O_2_ and malondialdehyde (MDA); this process causes membrane lipid peroxidation (Hu et al. 2016). Our previous study demonstrated that *S. oryzae* destroyed the metabolic balance and that K deficiency aggravated electrolyte leakage during the infection process (Zhang et al. 2019a, 2020). However, the metabolic mechanisms of host plants regulated by K nutrition to cope with *S. oryzae* infection are still imperfectly known.

Previous studies demonstrated that K deficiency promoted the biosynthesis of oxylipin (Troufflard et al. 2010; Armengaud et al. 2010). Furthermore, *S. oryzae* infection profoundly altered the hormone levels of the host plant, such as the content of abscisic and jasmonic acids (Peeters et al. 2020). Notably, lipid metabolism provides a substrate for the biosynthesis of jasmonic acid and oxylipids (Conconi et al. 1996; Torres-Franklin et al. 2009). Thus, exploring the whole metabolic profile of host plants at different K levels during *S. oryzae* infection could advance our understanding of the important role of K nutrition in rice against ShR disease. In this study, comparative metabolome and transcriptome analyses were performed to investigate the alterations in metabolites and transcriptome profiles between K-starved and K-sufficient rice during *S. oryzae* infection. The main objectives of this study were to select the predominant metabolic pathways regulated by K during *S. oryzae* infection and to explore the metabolic mechanisms of rice as regulated by K to cope with sheath rotting. Our findings provide comprehensive information on the metabolome, transcriptome, and ionome dynamics regulated by K during the infection process.

## Results

### Low-K Rice Leaf Sheaths are Hypersensitive to *S. oryzae* Infection

**Fig. 1:**
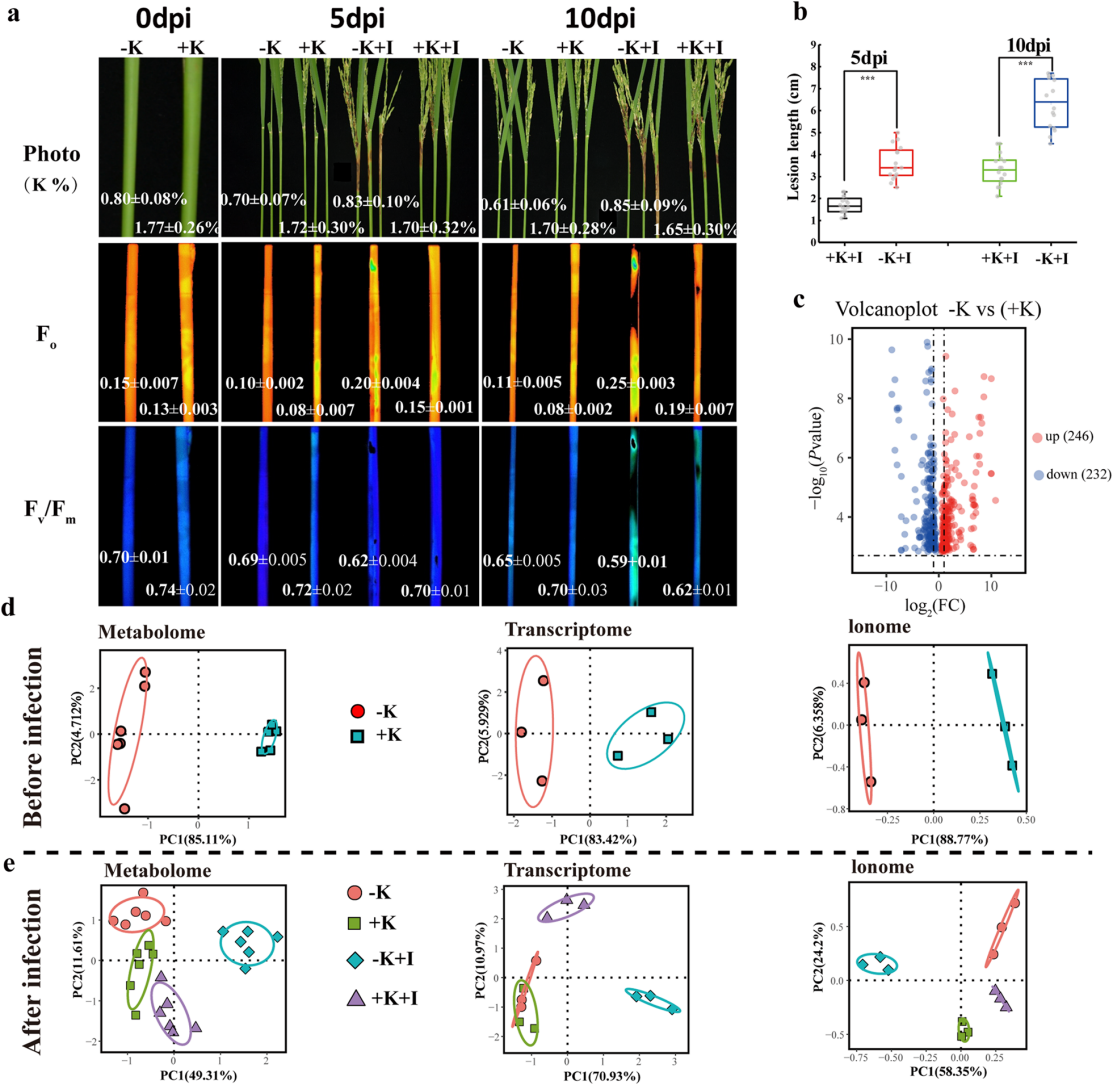
Rice deficiency in K shows hypersensitivity to *S. oryzae* infection. **a** The typical symptoms of ShR disease and the development of ShR among different K nutrition conditions. Numbers in the picture represent K concentrations, F_o_ and F_v_/F_m_ values; dpi denotes days post inoculation; the abbreviations of the treatments, K represent potassium, “+” and “−” denote with or without K; + I represent inoculation with *S. oryzae*. **b** Lesions lengths (n = 30 biologically independent tillers were selected for measurement, and five representative tillers were used for each replicate). **c** Volcano plots showing the up- and down-regulated genes in K-starved plants versus K-sufficient plants before inoculation. **d** Principal component analysis (PCA) of K-starved and K-sufficient FLS based on the corresponding metabolome, transcriptome, and ionome datasets before *S. oryzae* inoculation. The metabolite relative intensity values (n = 6 biological replicates), FPKM values for transcriptomes (n = 3 biological replicates), and elemental contents (n = 3 biological replicates) were used for PCA. **e** PCA of K-starved and K-sufficient FLS based on the corresponding metabolome, transcriptome, and ionome datasets at 5 days upon *S. oryzae* inoculation. Biological replicates for each treatment are outlined by the circles

The typical symptoms of ShR disease were observed in the FLSs upon *S. oryzae* inoculation. The minimum fluorescence (F_o_) was highest, while the maximum quantum efficiency of PSII photochemistry (F_v_/F_m_) was lowest in the − K + I treatment, which meant that K-starved rice was more sensitive to *S. oryzae* infection (Fig. 1a). The lesion length in the K-starved rice was significantly longer than that in the K-sufficient rice (Fig. 1b). Comparative metabolome and transcriptome analyses were used to examine the mechanisms underlying the further aggravation of *S. oryzae* infection by K deficiency. Before inoculation, 246 genes were significantly upregulated in the K-starved rice, and a fewer number of genes were downregulated (Fig. 1c). The K-starved rice differed significantly from the K-sufficient rice at the metabolic and transcription levels (Fig. 1d). Upon *S. oryzae* infection, principal component analysis clearly separated the infected and uninfected treatments by their metabolite profiles, transcripts, and ion contents. K starvation enhanced the differences in metabolites, transcripts, and ion contents between *S. oryzae* infected and uninfected FLSs (Fig. 1e).

### K Deficiency Induces Larger Alterations in the Predominant Metabolic Pathways Under *S. oryzae* Infection

**Fig. 2:**
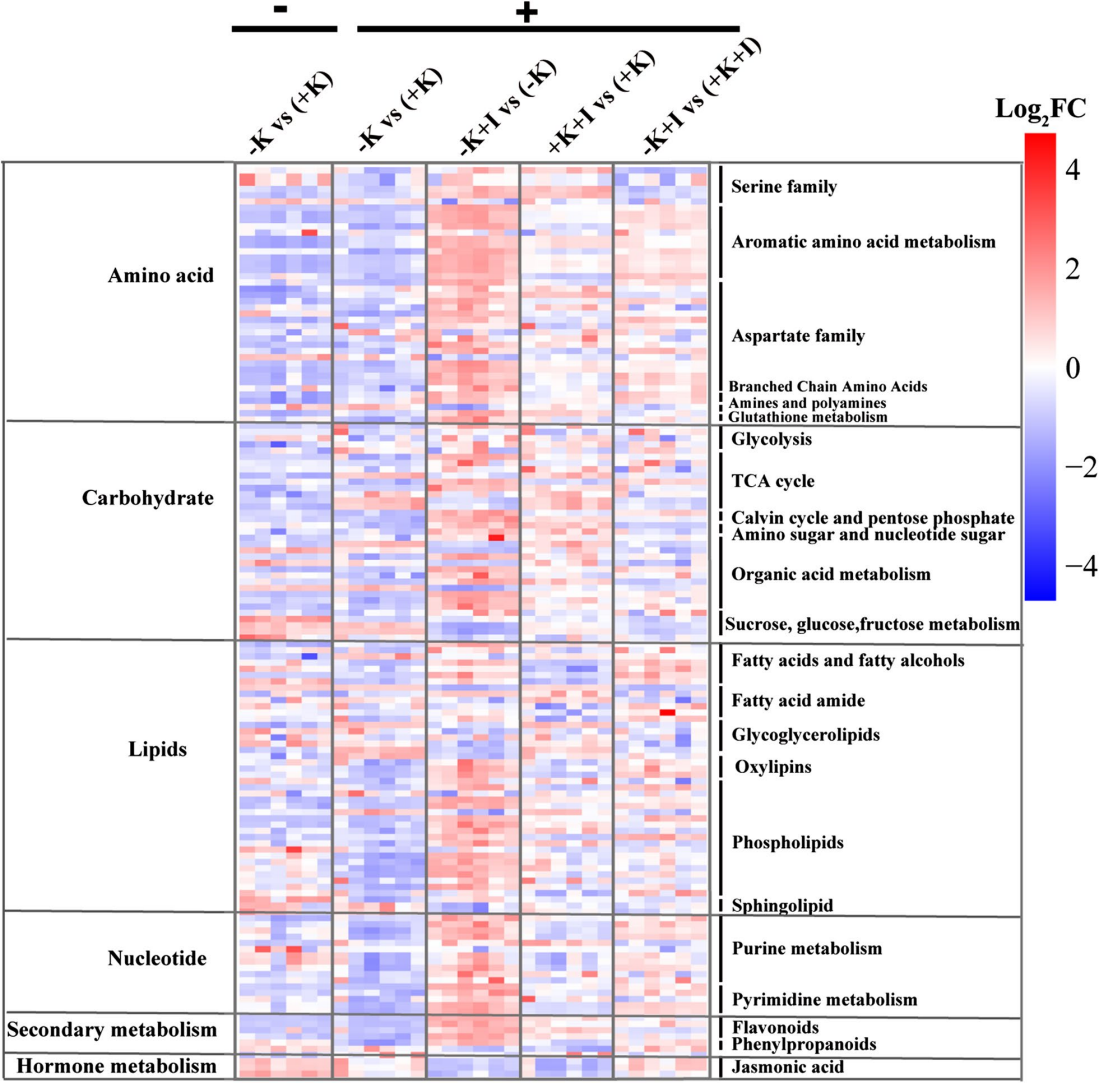
Infection with *S. oryzae* substantially altered metabolomes under K-starvation and K sufficient conditions, especially with respect to amino acids and lipid products. The data shown are log_2_(Fold Change) values of the metabolites (n = 6 biologically independent replicates), and the treatments in brackets were performed as controls. The 147 metabolites were clustered according to their metabolic pathways and functions, as suggested by previous study (McLoughlin et al. 2018). Additionally, *p*-value ≤ 0.05 and FDR ≤ 0.05 were set as the screening criteria for multiple comparisons. Red denotes higher log_2_(FC) values for the treatments, and blue denotes higher log_2_(FC) values for the controls (indicated in brackets). − and + indicate before inoculation and 5 days after inoculation (sterile water or *S. oryzae*), respectively

To assess how K deficiency aggravates *S. oryzae* infection by altering metabolic processes in the FLSs, we determined the quantitative profiles of 147 metabolites in the FLSs using nontargeted LC–MS/MS. Most of the metabolites related to amino acids, carbohydrates, lipids, nucleotides, secondary metabolism, and hormone metabolism were upregulated upon *S. oryzae* infection (Fig. 2).

**Fig. 3:**
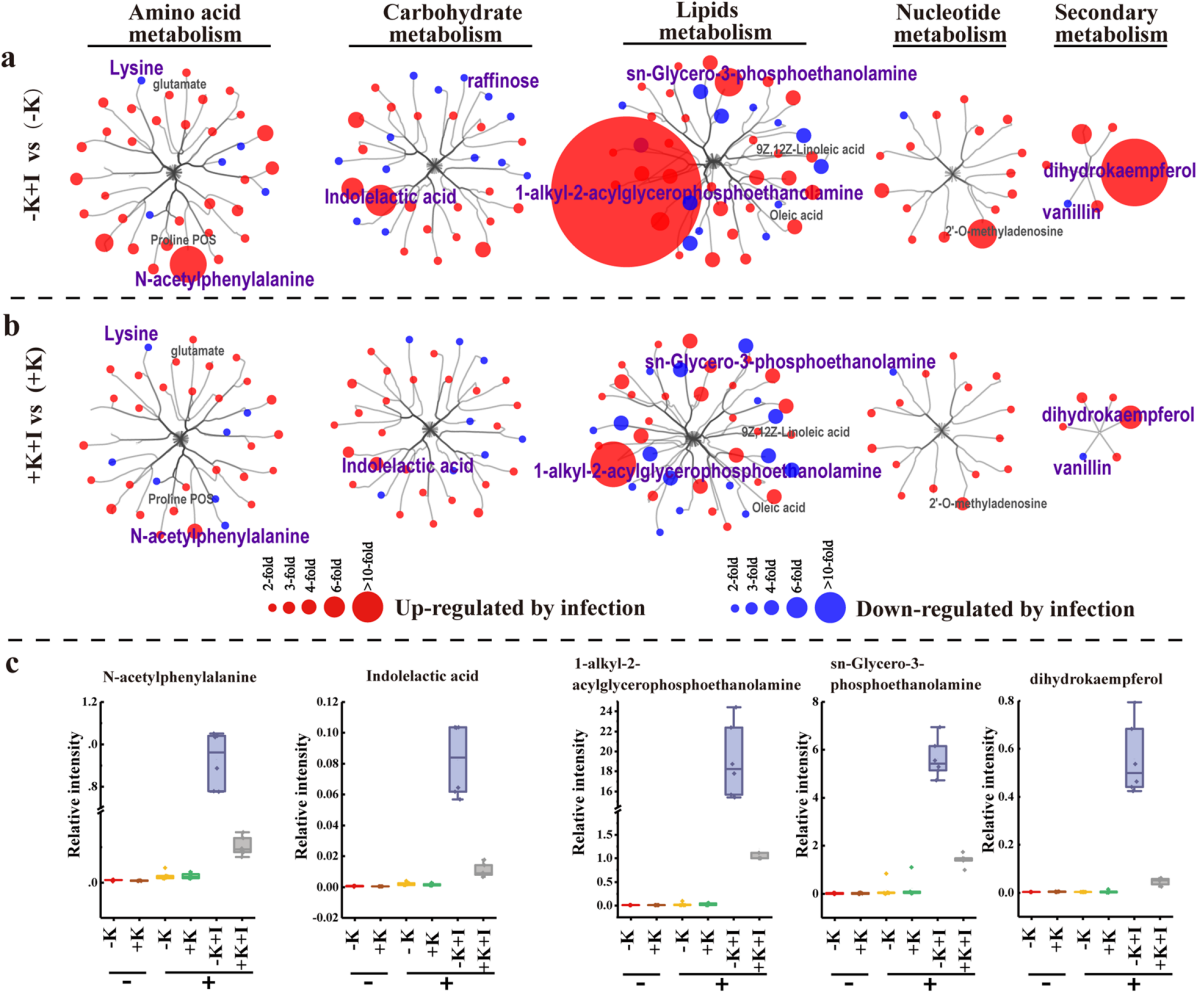
Survey of metabolic processes indicates that lipid metabolism is severely affected by *S. oryzae* infection. **a** Effects of *S. oryzae* infection on the metabolic profile of K-starved plants (at 5 days after inoculation). The metabolites were clustered using Cytoscape and the sizes of the circles denote the FC values (n = 6 biological replicates). **b** Effects of *S. oryzae* infection on the metabolic profile of K-sufficient plants. Blue indicates higher FC values for uninfected plants, and red denotes higher FC values for diseased plants; the treatments in brackets were performed as controls. **c** Examples of compounds substantially altered by *S. oryzae* infection. The center line in each box plot represents the mean relative intensity (n = 6)

We further organized all of the metabolites using Cytoscape software (Shannon et al. 2003). These compounds were clustered according to their function. The most obvious alterations were found in amino-acid metabolism and lipid metabolism (Fig. 3a, b). Lipid-breakdown products accumulated significantly in K-starved FLSs upon *S. oryzae* infection. For instance, the relative content of 1-alkyl-2-acylglycerophosphoethanolamine, a phospholipid, increased 18-fold in K-starved rice under *S. oryzae* infection compared to the corresponding value in the + K + I treatment (Fig. 3c). Other obvious changes in diseased FLSs were observed among compounds related to carbohydrate metabolism and secondary metabolism. For example, vanillin (belonging to secondary metabolism), which is associated with phenylpropanoid biosynthesis, was downregulated in the diseased rice (Fig. 3). The content of indoleacetic acid (IAA) in the diseased tissues was up-regulated, which consistent with a previous study reported that *S. oryzae* infection triggered strong IAA responses and IAA levels positively correlated with the lesion area (Peeters et al. 2020). Dihydrokaempferol is a derivative of flavonoids, the upregulation of which indicates that anthocyanin metabolism is activated.

### Metabolic and Transcriptomic Analyses Show That Membrane Lipid Peroxidation is Highly Induced by K Deficiency Under *S. oryzae* Infection

**Fig. 4:**
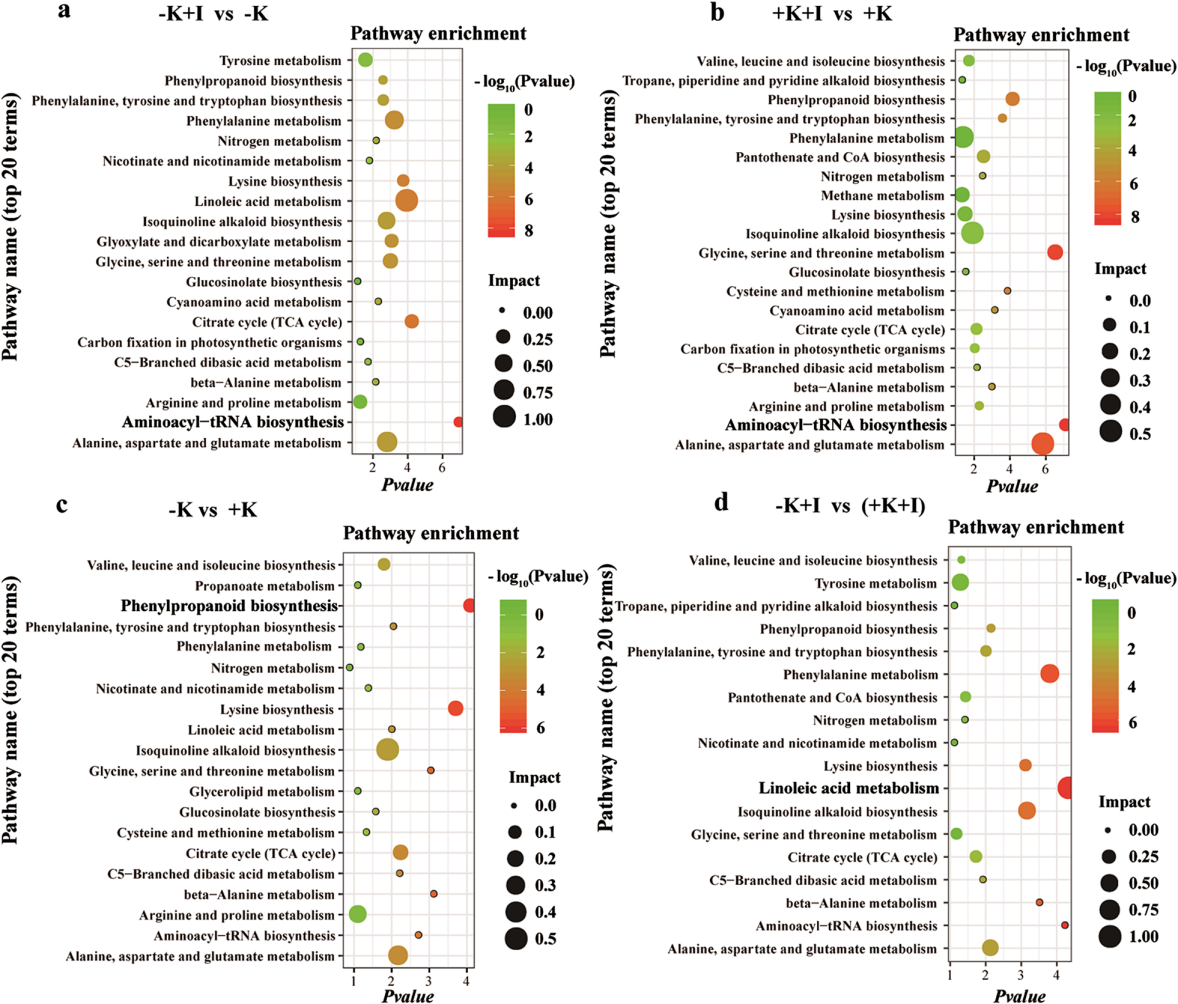
KEGG enrichment analysis of metabolites altered by *S. oryzae* infection. **a** The aminoacyl-tRNA biosynthesis pathway was dramatically enriched in K-starved plants after infection. **b** The aminoacyl-tRNA biosynthesis pathway was dramatically enriched in K-sufficient plants after infection. **c** The phenylpropanoid biosynthesis pathway was significantly enriched in K-starved plants versus K-sufficient plants before inoculation. **d** The linoleic acid metabolism pathway was dramatically enriched in K-starved plants versus K-sufficient plants upon *S. oryzae* infection. Orthogonal partial least squares discriminant analysis was used to evaluate the variable importance on the projection (size of the solid circle), and Student’s t-test (*p*-value < 0.05 denotes significance) was used to analyze the abundance of the metabolites. The color of the circle represents the − log_10_ (*p*-value)

Lysine and raffinose, which function as indicators of host plant tolerance to oxidative stress (Nishizawa et al. 2008), were down-regulated upon *S. oryzae* infection (Fig. 3). We combined metabolome analysis and Illumina-based RNA sequencing to further analyze alterations in the predominant metabolic pathways and gene expression profiles. All metabolites were annotated via the Kyoto Encyclopedia of Genes and Genomes (KEGG) analysis, top-20 significantly enriched pathways were selected based on *p*-values and impact factors (Fig. 4). The aminoacyl-tRNA biosynthesis pathway was significantly altered in the diseased FLSs, irrespective of K nutrition status (Fig. 4a, b). Before *S. oryzae* infection, K deficiency profoundly influenced the process of phenylpropanoid biosynthesis (Fig. 4c). However, *S. oryzae* infection increased the differences in linoleic acid metabolism between K-starved and K-sufficient rice (Fig. 4d).

**Fig. 5:**
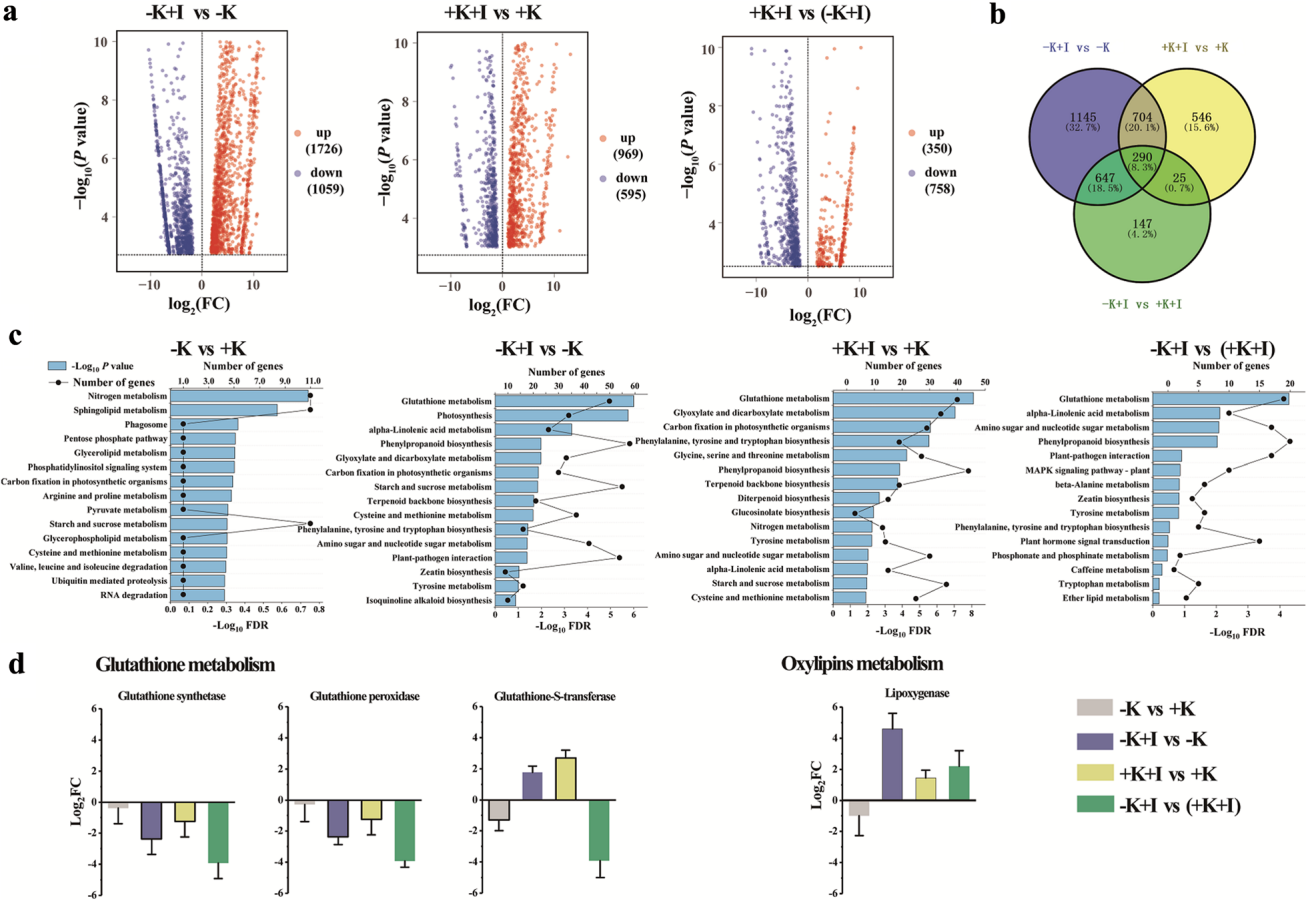
The rice transcriptome is significantly altered by *S. oryzae* infection under both K-starvation and K-rich conditions. **a** Volcano plots showing the up- and down-regulated genes in K-starved and K-sufficient rice upon *S. oryzae* infection. **b** Numbers of overlapped transcripts in diseased and healthy plants under K-rich and K-starvation conditions. **c** KEGG enrichment of DEGs between K-starved and K-sufficient rice under both infected and non-infected conditions. The glutathione and lipid metabolism pathways were obviously enriched in K-starved rice under *S. oryzae* infection. **d** Log_2_(FC) values of representative transcripts from specific KEGG pathways (see **c**). Treatments in brackets were performed as the controls

FPKM values were used to identify the differentially expressed genes (DEGs). Absolute values of log_2_ (FC) > 1.5, *p*-value < 0.01, and FDR < 0.05 were used to define DEGs. More DEGs were up-regulated in the K-deficient plants (1726 DEGs) than in the K-sufficient plants (969 DEGs) during infection (Fig. 5a). Independent of the K nutrition status of rice, 290 DEGs were found in the infected FLSs (Fig. 5b). Based on Gene Ontology (GO) annotation analyses, the DEGs were predominantly related to the membrane and membrane parts (Additional file 1: Fig. S1). As a response to oxidative stress and the damage to membrane parts caused by *S. oryzae* infection, the glutathione metabolism pathway was significantly enriched, as shown by KEGG pathway enrichment analysis of the DEGs (Fig. 5c). Additionally, upon *S. oryzae* infection, the representative transcripts for glutathione metabolism, such as the relative expressions of glutathione synthetase, glutathione peroxidase, and Glutathione-S-transferase, were up-regulated in K-sufficient plants compared the K-starved plants (Fig. 5d). By contrast, transcripts related to oxylipin metabolism, such as lipoxygenase were up-regulated in K-starved rice compared with K-sufficient rice upon *S. oryzae* infection (Fig. 5d). Most of the genes related to lipid metabolism, especially genes related to phospholipase D, were down-regulated in the K-deficient FLS after infection (Additional file 1: Table S2).

### K deficiency Amplified the Decrease in Galactolipids Content and Photosynthetic Capacity During *S. oryzae* Infection

**Fig. 6:**
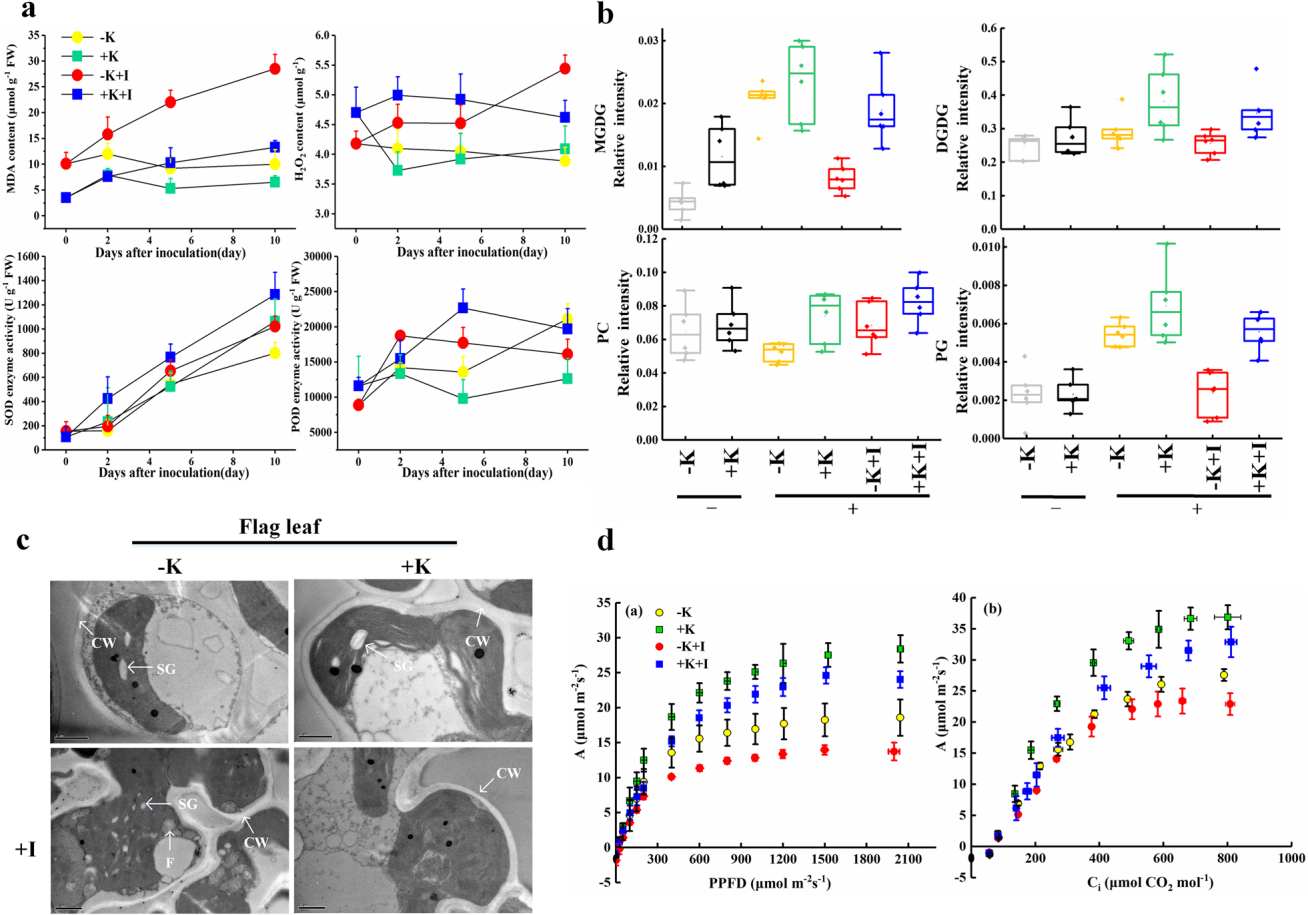
K deficiency aggravates membrane lipid peroxidation and induces a flag leaf response to *S. oryzae* infection. **a** MDA and H_2_O_2_ contents and activities of SOD and POD. The error bars on the top of the point denote the least significant difference among treatments at the same sampling date at *p* < 0.05; the abbreviations of the treatments, K represent potassium; I represent inoculation with *S. oryzae*; “+” and “−” represent with or without K, respectively. **b** The contents of MGDG, DGDG, PC, and PG; “−” and “+” under the treatments denote before and 5 days upon inoculation. **c** Ultrastructure of a chloroplast in the flag leaf. In the picture, CW, SG, and F represent the cell wall, starch grain, and a lipid droplet, respectively. **d** Light and CO_2_ response curves of the flag leaf

MDA is an indicator of membrane peroxidation, and K deficiency aggravated the accumulation of MDA in the FLSs during the infection process (Fig. 6a). Furthermore, *S. oryzae* infection can stimulate a burst of active oxygen. The content of H_2_O_2_ was up-regulated in K-sufficient plants within a short time from infection, followed a gradual stabilization. Conversely, K deficiency consistently induced the accumulation of H_2_O_2_ in the FLSs during infection. Likewise, K deficiency decreased the superoxide dismutase (SOD) and peroxidase (POD) activities during infection (Fig. 6a). Additionally, upon *S. oryzae* infection, K deficiency decreased the contents of MGDG, DGDG, phosphatidylcholine (PC) and phosphatidylglycerol (PG) by averages of 57.2%, 25.6%, 25.8%, and 57.6% respectively, in comparison with the + K + I treatment (Fig. 6b). Most phospholipid species, including PC and PG, were down-regulated in K-starved rice upon *S. oryzae* infection (Additional file 1: Table S3). As a systematic response of rice to *S. oryzae* infection, K deficiency altered the shape of the chloroplasts in the flag leaf (Additional file 1: Table S4). Likewise, lipid droplets occurred in K-starved plants during infection (Fig. 6c). The light and CO_2_ response curves showed that the photosynthetic potential of the flag leaf was inhibited by *S. oryzae* infection (Fig. 6d). K deficiency significantly decreased the net photosynthetic rate (*A*), maximum electron transport rate (*J*_*max*_), maximum carboxylation rate (*V*_*cmax*_), effective quantum efficiency of PSII (φPSII), and F_v_/F_m_ during *S. oryzae* infection in comparison with those in K-sufficient plants (Additional file 1: Table S5).

## Discussion

**Fig. 7:**
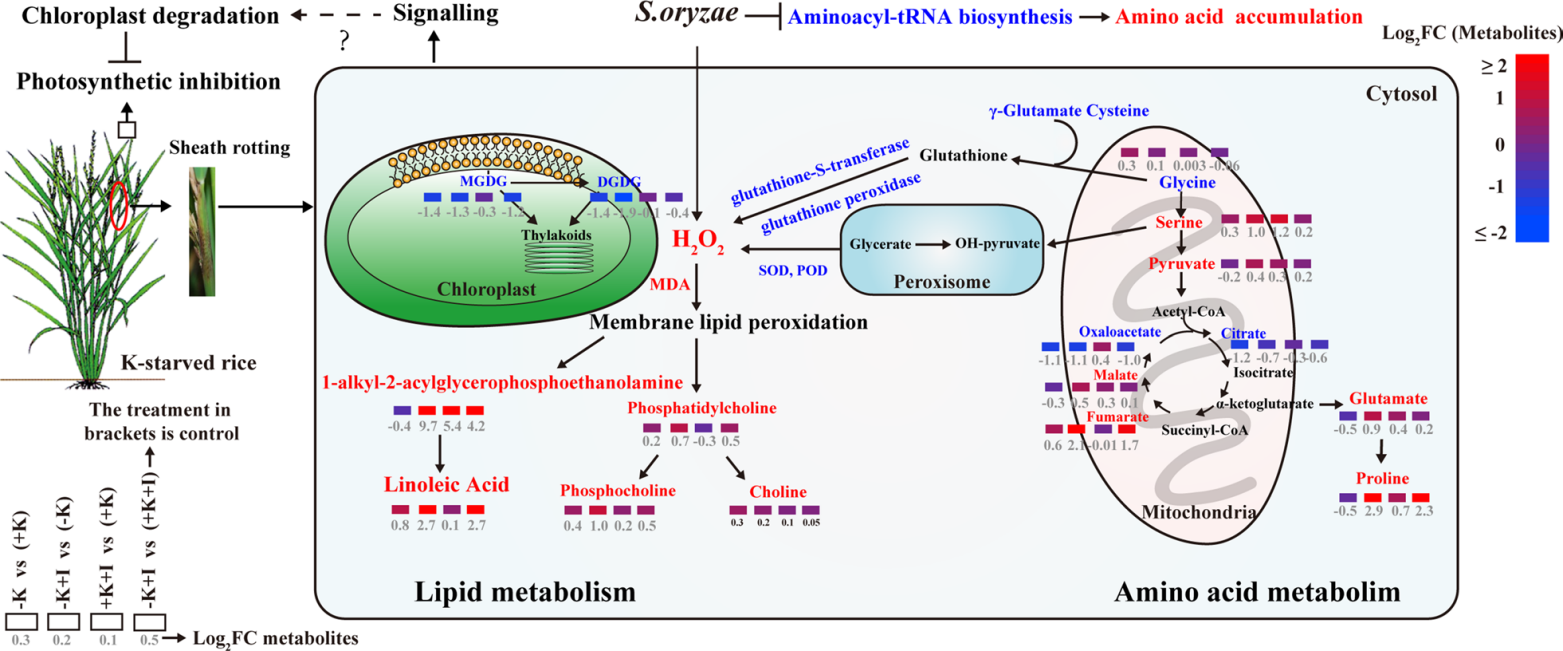
Summary model of the metabolic processes underlying the response of K-starved rice to *S. oryzae* infection. First, *S. oryzae* invades rice from FLSs and causes H_2_O_2_ accumulation, which results in severe oxidative stress. However, MGDG and DGDG contents, which are essential for maintaining membrane integrity and stability, were profoundly decreased in the K-starved FLSs. Likewise, K deficiency also decreases the synthesis of the antioxidant glutathione and the activities of SOD and POD. The consistent accumulation of H_2_O_2_ in K-starved rice induces lipid peroxidation, which accelerates 1-alkyl-2-acylglycerophosphoethanolamine and linoleic acid accumulation in K-starved FLSs. Second, *S. oryzae* infection impacts the biosynthesis of aminoacyl-tRNA, which results in amino acid accumulation in FLSs. Proline is upregulated and citrate is downregulated in K-starved rice, implying that energy metabolism and osmotic balance are disrupted by *S. oryzae* infection. As a systematic response, *S. oryzae* infection also induces the degradation of chloroplasts in flag leaves, which down regulates the photosynthetic rate of K-starved rice. Overall, alterations in these series of metabolic processes in K-starved rice promote *S. oryzae* development and sheath rotting. Note: FC in metabolite abundance for the treatments versus controls was calculated. The log_2_(FC) is indicated by the shade of red or blue according to the color scale, and the numbers below the rectangle indicate the corresponding log_2_(FC) values. The metabolites or physiological parameters in red/blue represent up/down regulation in − K + I-treated plants versus + K + I-treated plants. The symbol “?” means that the mechanism is not yet clear

K helps to improve crop tolerance to biotic stresses through different processes, such as by regulating the morphology, altering the metabolic profiles, and regulating ion homeostasis of the host plant (Amtmann et al. 2008; Shi et al. 2018). ShR disease caused by *S. oryzae* infection is a newly emerging fungal disease in rice with specific infection strategies (Bigirimana et al. 2015). However, the metabolic processes of the host plant regulated by K that allow it to cope with *S. oryzae* infection have seldom been revealed. The present study focused on the infection site (FLSs). We found that *S. oryzae* invades rice from FLSs and causes an H_2_O_2_ burst, which causes oxidative stress. However, K deficiency aggravated oxidative stresses and altered amino acid and lipid metabolism (Fig. 7). K starvation accelerated 1-alkyl-2-acylglycerophosphoethanolamine and linoleic acid hyperaccumulation, which further altered the lipid homeostasis of the host plant. In contrast, an adequate K supply decreased the accumulation of amino acid and lipid metabolism products by alleviating lipid peroxidation (Fig. 7). In the following, we discuss the effects of lipid peroxidation modulated by K on lipid metabolism and sheath rotting, followed by discussing the systematic responses of rice to *S. oryzae* infection under different K nutrition statuses.

### K Deficiency Alters Lipid Metabolism by Amplifying Lipid Peroxidation During *S. oryzae* Infection

Lipid metabolism involves the cell membrane process, which is crucial for rice–microbe interactions, and the peroxidation or degradation of the membrane contributes to cell death (Raffaele et al. 2009; Zoeller et al. 2012; Gao et al. 2017). However, in the present study, most glycerolipids including MGDG and DGDG, were downregulated upon *S. oryzae* infection (Fig. 2). In contrast, 1-alkyl-2-acylglycerophosphoethanolamine was hyperaccumulated in K-starved FLSs, altering the lipid homeostasis of the host plant. Fungal pathogen infection generally induces lipid peroxidation by causing oxidative stress (Zoeller et al. 2012). Invasion of cell tissues by *S. oryzae* can trigger a hypersensitivity response (HR) in the host plant. The characteristics of HR are NADPH oxidase initiation of an oxidative burst and production of O_2_^−^ at the cellular membrane (Torres et al. 2005). As the major source of reactive oxygen species (ROS) in the host plant, NADPH oxidase is found at relatively higher contents in K-starved plants (Shin and Schachtman 2004). Therefore, K starvation amplified oxidative stress in *S. oryzae-*infected tissues (Figs. 5c, 6a). Lipid peroxidation caused by excessive accumulation of ROS may result in cell membrane damage (Zoeller et al. 2012), which is consistent with our result that the MDA content (an indicator of membrane peroxidation) in K-starved rice was significantly higher than that in K-sufficient rice (Fig. 6a). Using trypan blue staining, we also visualized the ShR tissues, and the results indicated that the rate of cell death was much higher in the K-starved FLSs (Additional file 1: Fig. S2). Pathogen infection that induces lipid peroxidation exceeding a certain threshold level can contribute to membrane damage and cell death (Triantaphylidès et al. 2008). In this study, K deficiency significantly amplified lipid peroxidation and sheath rotting during *S. oryzae* infection. In contrast, the presence of sufficient K increased the biosynthesis of antioxidants (e.g., raffinose and glutathione) (Figs. 5c, d, 7), implying that a protective response is activated in K-sufficient rice to ameliorate the oxidative damage. This is consistent with a previous study indicating that sufficient K application significantly alleviates oxidative stress (Hu et al. 2016). In this study, glutathione transferase was significantly upregulated in K-sufficient rice (Fig. 5d), which mediated the stress tolerance of the host plant under biotic stress (Nianiou-Obeidat et al. 2017).

Additionally, lipid metabolism regulates plant–pathogen interactions predominantly through three strategies: the production of oxylipin or jasmonic acid (JA) in the lipoxygenase pathway, the reconstruction of membrane lipid defense signals in the unsaturated fatty acid pathway, and the synthesis of long-chain fatty acids (Raffaele et al. 2009). In the present study, glycoglycerolipids (MGDG and DGDG) and most phospholipids (PC, PG, and phosphatidylethanolamine) were downregulated in K-starved rice upon *S. oryzae* infection (Fig. 6b, Additional file 1: Table S3). This result is similar to that of a previous study reporting that *Magnaporthe oryzae* (fungal pathogen) infection significantly decreased the MGDG and DGDG contents (Zhou et al. 2008). However, some contrary results indicated that MGDG and DGDG contents in Arabidopsis were significantly upregulated by *Pseudomonas syringae* infection in a short time (24 h) (Zoeller et al. 2012). In our study, the downregulation of MGDG and DGDG was a strategy of *S. oryzae* for successful invasion of cell tissues. A previous study reported that lower levels of MGDG and DGDG can suppress immune responses in rice (Gao et al. 2017). In this study, we did not investigate the direct effects of MGDG and DGDG on *S. oryzae* colonization. However, a previous study demonstrated that a higher DGDG/MGDG ratio is an accommodative strategy for sustaining bilayer membranes under stress conditions (Perlikowski et al. 2016). Our results showed that the DGDG/MGDG ratio was significantly lower in K-starved rice (0.17) than in K-sufficient rice (0.31) during *S. oryzae* infection (Fig. 6b), which is in agreement with our hypothesis that K deficiency aggravates the degradation of the bilayer membrane during *S. oryzae* infection. Likewise, genes related to phospholipase D were downregulated by K deficiency during the infection process (Additional file 1: Table S2). The downregulation of phospholipase D activity might have been associated with remodeling or degradation of the lipid membrane (Adigun et al. 2021). Sufficient K supply significantly decreased the transcript expression of lipoxygenase (Fig. 5d), which might have suppressed the conversion of glycerolipids into oxylipins during *S. oryzae* infection. However, the content of JA in the FLSs did not increase (Fig. 2), which means that the conversion of linolenic acids into JA might have been suppressed. Likewise, *S. oryzae* infection produces cerulenin and helvolic acid, which destroy fatty acid metabolism and result in chlorosis symptoms, and the degradation of chloroplasts produces linoleic acid or linolenic acids in cell tissues as a response to environmental stress conditions (Conconi et al. 1996; Torres-Franklin et al. 2009; Bigirimana et al. 2015). These findings outline the possible reasons why linolenic acids and 1-alkyl-2-acylglycerophosphoethanolamine hyperaccumulated in K-starved FLSs during *S. oryzae* infection (Figs. 3a, 4d, 7). Changes in oleic acid levels are closely correlated with JA- triggered plant defense responses (Ruan et al. 2019). JA is initially synthesized from linolenic and linoleic acid. Thus, the hyperaccumulation of linoleic acid may suppress the JA level of the host plant, which is in agreement with a previous report that ShR infection slightly decreased the JA content of the host plant (Peeters et al. 2020). In the present study, we only investigated the effects of K starvation on lipid metabolism and lipid peroxidation during *S. oryzae* infection; however, other nutrient deficiencies might also induce membrane peroxidation, and whether other nutrient deficiencies could also promote ShR development needs further study.

### K Deficiency Alters Amino-Acid Metabolism and Photosynthetic Potential During *S. oryzae* Infection

In the present study, *S. oryzae* infection destroyed the integrity and stability of cell membranes. Infection with *S. oryzae* results in the secretion of phytotoxin into cells (Hittalmani et al. 2016), which may suppress the biosynthesis of aminoacyl-tRNA (Fig. 4a, b). Because aminoacyl-tRNA is used for amino acid transfer into the ribosome for protein synthesis (Duchêne et al. 2005; Sheppard et al. 2008), its suppression resulted in amino acid hyperaccumulation in the FLSs (Fig. 2). K deficiency aggravated the accumulation of amino acids in the FLSs and further altered the osmotic characteristics and mineral nutrient homeostasis of the host cells (Additional file 1: Fig. S3). The accumulation of amino acids and organic acids in the FLSs decreased the pH of the cell microenvironment, and the consistent accumulation of H_2_O_2_ at the infection site(s) supported this result (Fig. 6a). As K^+^ is essential for chloroplast osmoregulation and integrity regulation, an increasing K^+^ concentration regulates the pH of the cell microenvironment by offsetting the H^+^ concentration (Kunz et al. 2014). Maintaining a proper pH in the cell microenvironment is a feasible strategy for the host plant against fungal pathogen infection (Robison et al. 2018). Thus, in the present study, the K-starved rice exhibited an increasing trend of K^+^ at the infection site (Additional file 1: Fig. S3). However, the increased K^+^ content in the K-starved FLSs was at the expense of decreasing K^+^ content at uninfected site(s), such as stems (Additional file 1: Fig. S4). In this study, most of the elemental contents were diminished slightly with an extension of the infection time, which was due mainly to the exportation of elements from the FLSs to the grains due to grain-filling. However, K deficiency delayed the exportation of most elements from the infected FLSs (Additional file 1: Fig. S3), possibly due to the destroyed cell membranes inhibiting the phloem-loading process at the infection site(s) (Chen 2014). Additionally, as a systematic response, fungal pathogen infection induced the HR process, which reduced the photosynthetic rate (Berger et al. 2007). This is consistent with a previous study showing that progressive deformation of the chloroplast leads to a diminished photosynthetic rate (Fig. 6c, d), which is a consequence of the systemic response to *S. oryzae* infection. K starvation decreased the systemic tolerance capacity of the host rice by enhancing the negative impacts of *S. oryzae* infection on leaf photosynthetic potential.

## Conclusions

*S. oryzae* infection induces amino-acid accumulation in FLSs by impacting aminoacyl-tRNA biosynthesis. During the infection process, K starvation sharply upregulated the 1-alkyl-2-acylglycerophosphoethanolamine content but decreased the glycoglycerolipids content, which altered lipid metabolism and destroyed lipid homeostasis. K deficiency in rice induced H_2_O_2_ hyperaccumulation in diseased tissues, which caused lipid peroxidation. In contrast, a sufficient K supply increased antioxidant biosynthesis and activated SOD and POD enzyme activities, which alleviated the oxidative stress caused by *S. oryzae* infection. K deficiency decreased the photosynthetic potential of the flag leaf by altering the morphology of chloroplasts during *S. oryzae* infection. Overall, these results emphasize the important role of K in alleviating *S. oryzae* infection by regulating amino acid and lipid metabolism.

## Materials and Methods

### Plant Materials and Growth Conditions

Rice seeds (*O. sativa* cv. Shengliangyou 5814, Indica rice) were disinfected with 0.5% NaClO for 10 h, followed by germination in deionized water at 30 °C for 3 days. After germination, seeds were moved to a plastic net, which was fixed on a foam material and floated in deionized water. Seven days later, the uniform seedlings were transplanted to a container (6 L) for 8 days (50% strength nutrient solution for 3 days and 100% strength nutrient solution for 5 days). Finally, 15-day old seedlings were used for a pot experiment. The soil material used for the pot experiment was collected from a long-term K fertilizer field experiment in Wuxue County (30°06′46″ N, 115° 36′9″ E), Hubei Province, central China. The soil chemical properties were as follows: total nitrogen, 1.8 g kg^−1^; Olsen-phosphorus (P), 13.0 mg kg^−1^; available K, 30.0 mg kg^−1^; slowly available K, 288 mg kg^−1^; and organic matter, 34.0 g kg^−1^. The soil type was sandy loam soil with a pH of 5.60 (1:2.5, soil: deionized water), which was categorized as K-deficient soil (Zou et al. 2011). The pot experiment was conducted in a greenhouse with day/night temperatures of 28/22 °C, a relative humidity of 45–55%, and a photon flux density of 800 μmol m^−2^ s^−1^.

### Experimental Design and *S. oryzae* Inoculation

Two K levels were used in the pot experiment, which was performed with 12 replicates. In total 24 pots were used. The treatments used were (1) sufficient K supply (+ K), with an application rate of 1.5 g K_2_O/kg soil (in the form of potassium sulfate), and (2) zero K application (-K), with no K fertilizer applied to the pot. To ensure that other nutrients were not limiting factors for rice growth, 2.0 g N/kg soil (in the form of urea) and 1.5 g P_2_O_5_/kg soil (in the form of calcium superphosphate) were applied as basal fertilizers 1 day before transplanting. All fertilizers were mixed with the soil (total of 10 kg soil/pot) before watering. Three single seedlings were transplanted into each pot.

Pathogen material (*S. oryzae*) was isolated from diseased plants in the field, as described in our previous study (Zhang et al. 2019a). Sixty-day-old plants (at the booting stage) were used for pathogen inoculation. First, *S. oryzae* was incubated on potato dextrose agar medium, which was placed in an incubator at 28 °C for 8 days. Immediately afterward, sterile water was added to the plate-grown mycelium to leach a conidial suspension. For inoculation, uniform FLSs in the − K and + K treatments were selected for inoculation with *S. oryzae* at a concentration of 1 × 10^7^ conidia mL^−1^. For each treatment, six pots were selected for inoculation. Briefly, a punch was used to make a small hole in the leaf sheath at the same position for each leaf sheath, and then 10 μL of suspended spores was injected. The control plants were injected with sterile water (10 μL) as mock inoculation. Next, all pots were placed in darkness with 90% humidity for 12 h. After successful infection, the following treatments were prepared: (1) K-starved plants with mock inoculation (− K), (2) K-sufficient plants with mock inoculation (+ K), (3) K-starved plants with *S. oryzae* inoculation (− K + I), and (4) K-sufficient plants with *S. oryzae* inoculation (+ K + I).

### Metabolome Profiling and Data Analysis

Twelve biological replicates of fresh FLS tissues (five representative FLSs were mixed as one sample) were sampled before inoculation (− K and + K) and at 5 days after inoculation (− K, + K, − K + I, + K + I). All samples were frozen in liquid nitrogen, and then stored at − 80 °C for extraction of metabolites, lipids, and RNA. Metabolite extraction followed the method described by with some modifications (Kumar et al. 2015). Briefly, 50 mg of fresh sample was extracted with 1000 μL of solvent (acetonitrile: methanol: water, 2:2:1, containing 1 μg/mL of internal standard). This process was repeated three times. Then, incubation at − 20 °C for 1 h and centrifugation at 4 °C and 12,000 rpm for 15 min were performed. The supernatant was stored in vial at − 80 °C until further use. Liquid chromatography with tandem mass spectrometry (LC–MS/MS) analyses were performed using an ultra-high performance liquid chromatography system (1290, Agilent Technologies, Santa Clara, CA, USA) with a UPLC HSS T3 column (2.1 mm × 100 mm, 1.8 μm, Waters, Tokyo, Japan) coupled to a Q Exactive system. An accurate quadrupole time-of-flight mass spectrometer (AB Triple TOF 6600, AB Sciex, Framingham, MA, USA) was used for all mass spectrometry measurements. Metabolites were identified by comparing the ion features and retention times to the reference chemical library (DeHaven et al. 2010). Student’s t-test was used to determine the significance of differences in each metabolite among the *S. oryzae*-inoculated and mock-inoculated samples. The KEGG (http://www.kegg.com) database was used for annotation of the metabolites (Additional file 2: Metabolites data). Additionally, classification of metabolites was performed using the method suggested by a previous study (McLoughlin et al. 2018).

### Transcriptome Profiling

Fresh samples were collected before inoculation and at 5 days after inoculation. Total RNA extraction was performed using TRIzol reagent (Invitrogen, Carlsbad, CA, USA) according to the manufacturer’s instructions, followed by polyA selection for mRNA enrichment. RNA sequencing was performed by Majorbio Bio-pharm Technology Co. (Shanghai, China) using the Illumina Hiseq-2500 sequencing system (San Diego, CA, USA). The sequence data were mapped to the *Oryza sativa Indica Group* reference genome (ASM4651v1) (Additional file 1: Table S1) from the Ensembl Plants database (http://plants.ensembl.org/Oryza_indica/Info/Index) using TopHat2 software (Kim et al. 2013). The DEGs were identified using edgeR software (version 3.24.3). We calculated the gene expression level using fragments per kilobases per million reads (FPKM, Additional file 3: FPKM values) (Mortazavi et al. 2008). To identify the DEGs between the controls and treatments, we set a false discovery rate (FDR) ≤ 0.05, absolute log_2_(fold change (FC)) ≥ 1.5, and *p*-value ≤ 0.01 as the discriminant standards. KOBAS2.0 was used to analyze the KEGG pathway enrichment of the DEGs (Xie et al. 2011).

### Ionome Profiling

Samples used for measurements of elemental contents were collected at 0, 2, 5, and 10 days after *S. oryzae* inoculation. Sixteen elements were measured via inductively coupled plasma mass spectrometry (ICP-MS) using the method suggested by a previous study (McLoughlin et al. 2018). Briefly, 0.1 g of dry sample was digested in a mixture of HNO_3_ and HClO_4_ (4 HNO_3_:1 HClO_4_), and the digested solution was then dissolved in 50 ml of ultra-pure water. After filtering with double filter paper, this solution was used for ICP-MS profiling. A multi-element standard (Ultra Scientific, Providence, RI, USA) was used for calibration curves to monitor the run-to-run variation.

### MDA, H_2_O_2_, Superoxide Dismutase, Peroxidase, and Lipid Extraction Assays

MDA, H_2_O_2_ content, and SOD and POD enzyme activities were measured according to the method described by Djanaguiraman et al (2009). Fresh FLSs sampled before and at 5 days after *S. oryzae* inoculation were used for lipid extraction. The lipid content measurements were performed using electrospray ionization tandem mass spectrometry (ESI–MS/MS) according to the method suggested by a previous study with some modifications (Djanaguiraman et al. 2009; Liu et al. 2015). Briefly, to inactivate phospholipase activity, samples were placed in tubes containing 3.0 ml isopropanol with 0.01% butylated hydroxytoluene (BHT) for 20 min, followed by extraction with 1.5 ml chloroform and 0.5 ml water for 1.5 h. Then, the samples were extracted with 4.0 ml chloroform/methanol (2:1, v/v) containing 0.01% BHT. This extraction step was repeated six times. Finally, the lipid extracts were washed with 1 mM KCl and sterile water. Nitrogen gas was used to dry the lipid phases. Then, the extracts were dissolved in chloroform and used for ESI–MS/MS analysis. The remaining FLS tissues were dried in an oven, and the lipid content was calculated based on the dry weight. Automated ESI–MS/MS was used for lipid analysis. The approach and data processing followed a previously described method, and internal standards were used for the calculation of lipid contents (Narayanan et al. 2016). MGDG, DGDG, PC and PG contents were measured independently with six biological replicates.

### Chlorophyll Fluorescence Imaging, Photosynthetic Rate, and Chloroplast Ultrastructure

Chlorophyll fluorescence imaging was performed using the MINI-Version Imaging-PAM (IMAG-MIN/B, Walz, Effeltrich, Germany) system following the method suggested by Lu et al. (2017). At 5 days upon inoculation, the flag leaf was selected for gas-exchange measurements, which were performed using a portable photosynthesis system (Li-6400XT, Li-Cor, Inc., Lincoln, NE, USA) from 9:00 to 11:30 a.m. Leaves used for light and CO_2_ response-curve measurements were previously acclimated to saturating light for 30 min. Thirteen levels of photosynthetic photon flux density (PPFD) (2000, 1500, 1200, 1000, 800, 600, 400, 200, 150, 100, 50, 25, and 0 μmol m^−2^ s^−1^) were used to determine the light-response curve. Additionally, a series of CO_2_ concentrations (400, 300, 200, 100, 50, 400, 600, 800, 1000, 1200, and 1500 μmol CO_2_ mol^−1^) was used to determine the CO_2_ response curve at a constant PPFD of 1500 μmol m^−2^ s^−1^. After the curve measurements, flag leaf and FLS segments were sampled for ultrastructural observations following the method described by Lu et al. (2016).

### Statistical Analysis

Data analysis was performed using the least significant difference test with α = 0.05 in SPSS 19.0 (SPSS, Inc., Chicago, IL, USA). Figures were drawn using Origin 9.0 software (OriginLab Corporation, Northampton, MA, USA) and R3.6.1 (R Foundation for Statistical Computing, Vienna, Austria) with the “pheatmap” package.

## Supplementary Information

**Additional file 1: Fig. S1** GO function classification of DEGs. **Fig. S2.** Trypan blue staining of FLSs. **Fig. S3** K deficiency alters the elemental contents of FLS during S. oryzae infection. **Fig. S4** K concentrations of healthy and diseased plants among different organs. **Table S1** Mapping statistics of K-starved rice and K-sufficient rice at 0 and 5 days post inoculation. **Table S2** Expression profile of lipid metabolism related genes in the FLS. **Table S3** Lipid species related to phospholipids metabolism based on the top 10 VIP scores. **Table S4** Morphological parameters of chloroplasts. **Table S5** Photosynthetic characteristics of flag leaf.

**Additional file 2.** Metabolites data.

**Additional file 3.** FPKM values.

## Data Availability

The datasets used and/or analysed during the current study are available from the corresponding author on reasonable request.
