# Supplementary material for: Metabolomic and Transcriptomic Changes Induced by Potassium Deficiency During Sarocladium oryzae Infection Reveal Insights into Rice Sheath Rot Disease Resistance
Source: Rice (N Y). 2021 Sep 17;14:81. doi: 10.1186/s12284-021-00524-6 (PMC8448798; doi:10.1186/s12284-021-00524-6)
Supplement: Supplementary file 1 — Additional file 1: Fig. S1 GO function classification of DEGs. Fig. S2. Trypan blue staining of FLSs. Fig. S3 K deficiency alters the elemental contents of FLS during S. oryzae infection. Fig. S4 K concentrations of healthy and diseased plants among different organs. Table S1 Mapping statistics of K-starved rice and K-sufficient rice at 0 and 5 days post inoculation. Table S2 Expression profile of lipid metabolism related genes in the FLS. Table S3 Lipid species related to phospholipids metabolism based on the top 10 VIP scores. Table S4 Morphological parameters of chloroplasts. Table S5 Photosynthetic characteristics of flag leaf. [file 12284_2021_524_MOESM1_ESM.docx]

**Supplementary data for:**

**Metabolomic and transcriptomic changes induced by potassium deficiency during *Sarocladium oryzae* infection reveal insights into rice sheath rot disease resistance**

Jianglin Zhang^a^, Zhifeng Lu^a^, Tao Ren^a^, Rihuan Cong^a^, Jianwei Lu^a^, Xiaokun Li^a*^

*^a^* *Key Laboratory of Arable Land Conservation (Middle and Lower Reaches of Yangtze River), Ministry of Agriculture and Rural Affairs, P. R. China, Wuhan 430070, China; Microelement Research Center, Huazhong Agricultural University, Wuhan 430070, China.*


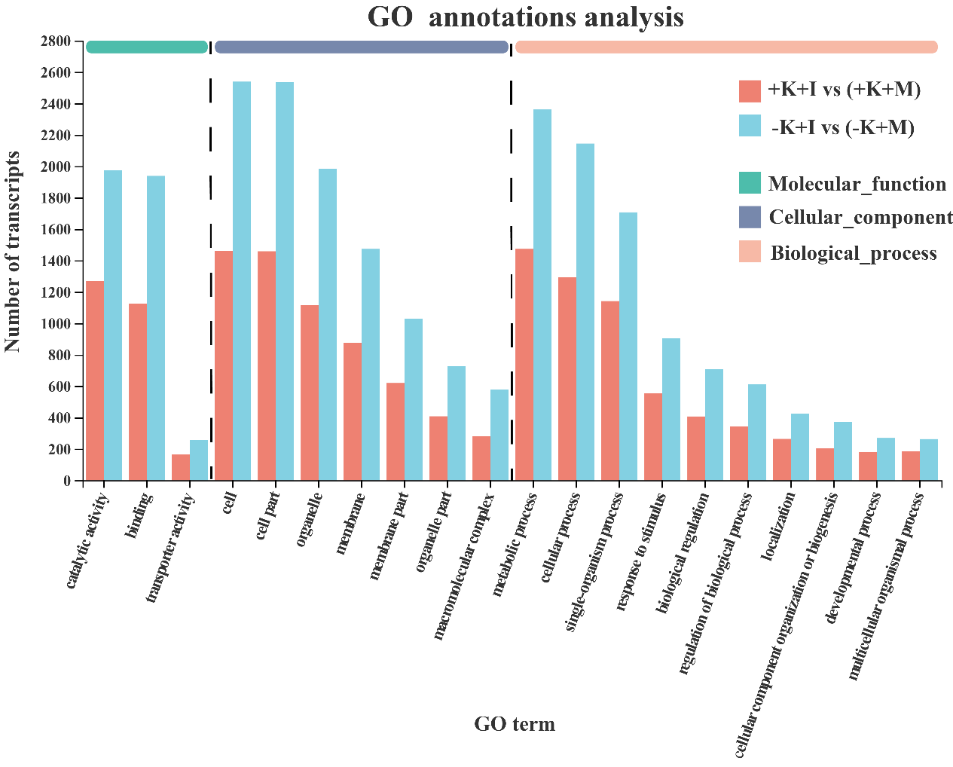


**Fig S1** Gene ontology (GO) terms associated with differentially expressed genes (DEGs) in infected FLS versus uninfected FLS. Terms were grouped into the following three categories: molecular functions (green); cellular components (blue) and biological processes (red). Differences in percentages were considered significant at *P* ≤ 0.05. Most of the DEGs were associated with membrane and membrane part in cellular component.


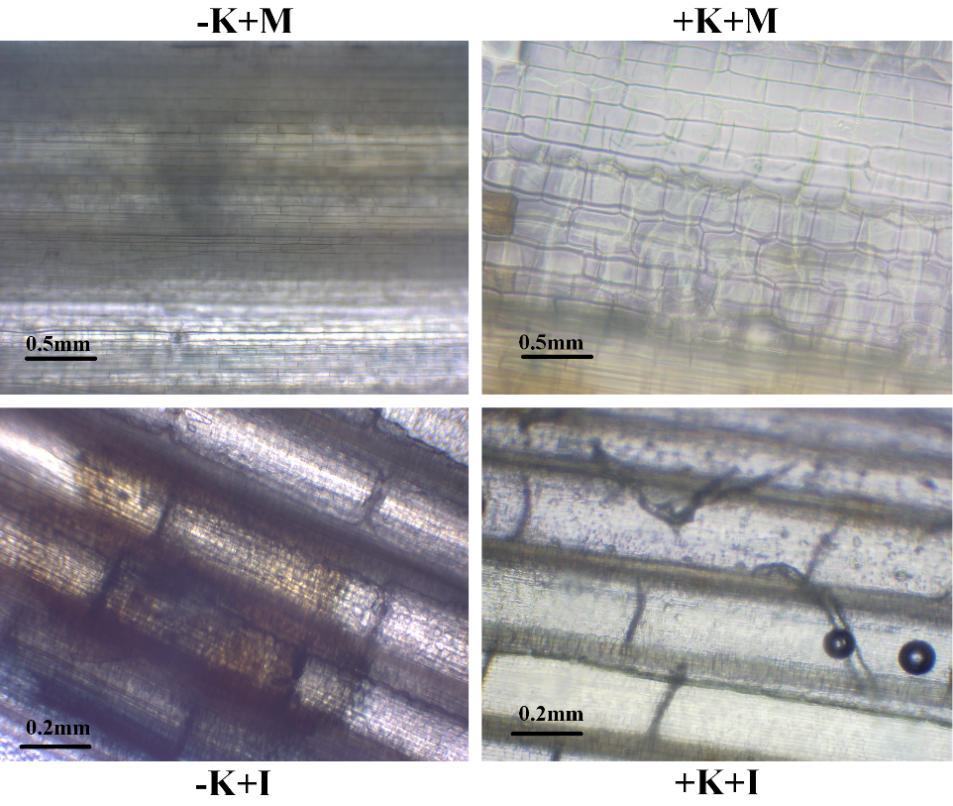


**Fig S2** Cell death of flag leaf sheath caused by *S. oryzae* infection.

**Note:** Cell death induced by *S. oryzae* infection was visualized by trypan blue staining at 2 days post inoculation (inner epidermis). The dark blue implying the cell death and the staining process was followed the method suggested by Bai et al. (2012).





**Fig S3** K deficiency alters the elemental contents and ion homeostasis in the FLS during S. oryzae infection.

Using ICP-MS, 16 commonly found elements were quantified based on the time series after S. oryzae infection. Most of the elements content were diminished slightly in uninfected-FLS during the grain-filling process. However, during S. oryzae infection, the contents of K, manganese (Mn) and nickel increased slightly in the K-starved FLS compared to the corresponding values in the +K+I treatment. Infection with S. oryzae infection delayed the exportation of K, copper, magnesium (Mg) and P from infected FLSs during the grain-filling process. However, K deficiency induced the accumulation of Mn in the FLS during S. oryzae infection. It is noteworthy that S. oryzae infection slightly enhanced the accumulation of K in the FLSs of K-starved rice; conversely, the content of K in K-sufficient FLSs slowly decreased.





**Fig S4** Potassium concentrations of the healthy and diseased plants among different organs under four K application levels. Based on the field experiment, K_0_, K_60_, K_120_, K_180_ denotes 0, 60,120 and 180 kg K_2_O ha^-1^application, respectively. Diseased represent rice infection with *S. oryzae*.

**Table S1** Mapping statistics of K-starved rice and K-sufficient rice at 0 and 5 days post inoculation.

| **Sample ID** | **Total reads count** | **Total mapped reads** | **Multiple mapped reads, n(%)** | **Uniquely mapped reads, n (%)** | **Overall mapping (%)** |
| --- | --- | --- | --- | --- | --- |
| -K_1 | 54120300 | 51961399 | 2761254(5.1%) | 49200145(90.91%) | 96.01% |
| -K_2 | 50446498 | 48625008 | 3013898(5.97%) | 45611110(90.41%) | 96.39% |
| -K_3 | 56113124 | 53997415 | 3418565(6.09%) | 50578850(90.14%) | 96.23% |
| +K_1 | 54629692 | 52562444 | 5575673(10.21%) | 46986771(86.01%) | 96.22% |
| +K_2 | 53209714 | 51151046 | 3474532(6.53%) | 47676514(89.6%) | 96.13% |
| +K_3 | 52272690 | 50175532 | 3514788(6.72%) | 46660744(89.26%) | 95.99% |
| -K+M_1 | 52030508 | 46724749 | 3772024(7.25%) | 42952725(82.55%) | 89.80% |
| -K+M_2 | 45344364 | 42798491 | 2673057(5.9%) | 40125434(88.49%) | 94.39% |
| -K+M_3 | 48171162 | 45665275 | 3967014(8.24%) | 41698261(86.56%) | 94.80% |
| +K+M_1 | 48964240 | 46643156 | 4660629(9.52%) | 41982527(85.74%) | 95.26% |
| +K+M_2 | 49083346 | 46104033 | 5365622(10.93%) | 40738411(83.0%) | 93.93% |
| +K+M_3 | 57321494 | 53421038 | 4268775(7.45%) | 49152263(85.75%) | 93.20% |
| -K+I_1 | 49215888 | 30191700 | 1472719(2.99%) | 28718981(58.35%) | 61.40% |
| -K+I_2 | 45347356 | 31538943 | 2385262(5.26%) | 30153681(66.49%) | 69.50% |
| -K+I_3 | 48042816 | 42977172 | 2976925(6.2%) | 40000247(83.26%) | 89.46% |
| +K+I_1 | 47348774 | 44488878 | 2646787(5.59%) | 41842091(88.37%) | 93.96% |
| +K+I_2 | 53350418 | 41602906 | 3119335(5.85%) | 38483571(72.13%) | 77.98% |
| +K+I_3 | 54885760 | 38096138 | 3192192(5.82%) | 34903946(63.59%) | 69.41% |

**Table S2** Expression profile of lipid metabolism related genes in the FLS before and after *S. oryzae* inoculation under different K nutrition status.

| **Gene ID** | **Gene description** | **Fold change (log_2_)** | | | |
| --- | --- | --- | --- | --- | --- |
|  |  | **-K+M vs (+K+M)** | **-K+I vs (-K+M)** | **+K+I vs**  **(+K+M)** | **-K+I vs**  **(+K+I)** |
| BGIOSGA031184 | Phospholipase D | 0.40 | -1.99 | 0.54 | -2.14 |
| BGIOSGA020106 | Glycerol-3-phosphate dehydrogenase | 0.29 | -0.24 | 1.01 | -0.97 |
| BGIOSGA000693 | Diacylglycerol kinase | 0.58 | -0.38 | 1.55 | -1.36 |
| BGIOSGA001799 | Phospholipase | -0.33 | -1.51 | -0.92 | -0.92 |
| BGIOSGA040585 | FAD-dependent oxidoreductase family protein | 0.48 | -0.34 | 0.62 | -0.47 |
| BGIOSGA033204 | 1-acyl-sn-glycerol-3-phosphate acyltransferase | -0.13 | -1.49 | -0.07 | -1.55 |
| BGIOSGA005137 | Glycerol-3-phosphate dehydrogenase | 0.41 | -1.08 | -1.23 | 0.56 |
| BGIOSGA026396 | Alpha-galactosidase | 0.55 | 0.58 | 2.11 | -0.98 |
| BGIOSGA023223 | Phospholipase D | -0.52 | -1.04 | 0.05 | -1.61 |
| BGIOSGA035954 | Diacylglycerol kinase | 0.17 | -1.01 | -0.01 | -0.83 |
| BGIOSGA008719 | Aldehyde dehydrogenase | 0.56 | -0.93 | -0.27 | -0.10 |
| BGIOSGA011652 | Phosphatidylserine decarboxylase proenzyme 1, mitochondrial | 0.05 | -1.15 | -0.07 | -1.03 |
| BGIOSGA019324 | O-acyltransferase | 0.13 | -1.76 | -0.92 | -0.71 |
| BGIOSGA023222 | Phospholipase D | 0.38 | -2.59 | -1.27 | -0.95 |
| BGIOSGA035060 | Non-lysosomal glucosylceramidase | 0.62 | -0.91 | 0.52 | -0.81 |
| BGIOSGA018725 | Phospholipase D | -0.39 | -2.03 | -1.37 | -1.06 |
| BGIOSGA037093 | Aldehyde dehydrogenase | 0.27 | -2.73 | -0.89 | -1.56 |
| BGIOSGA017654 | Beta-galactosidase | 0.28 | -0.46 | -0.04 | -0.15 |
| BGIOSGA000642 | Glycerol-3-phosphate dehydrogenase | 0.53 | 1.83 | 1.38 | 0.97 |
| BGIOSGA024503 | Phospholipase D | 0.34 | -2.10 | -0.45 | -1.32 |
| BGIOSGA008611 | Phosphatidyl-N-methylethanolamine N-methyltransferase | 0.00 | -0.33 | 0.35 | -0.68 |
| BGIOSGA007213 | CDP-diacylglycerol-inositol 3-phosphatidyltransferase | -0.41 | -0.17 | 0.39 | -0.97 |
| BGIOSGA017928 | Aldo-keto reductase | 0.57 | -1.42 | -1.07 | 0.22 |
| BGIOSGA012913 | Diacylglycerol kinase | 0.27 | -2.29 | -0.65 | -1.38 |
| BGIOSGA033188 | Alpha-galactosidase | 0.21 | -1.05 | 0.09 | -0.93 |
| BGIOSGA027553 | Diacylglycerol kinase | 0.07 | -0.26 | -0.03 | -0.16 |
| BGIOSGA031703 | Non-lysosomal glucosylceramidase | 0.33 | -1.81 | -0.50 | -0.98 |

**Note:** Gene description corresponds to putative function as described in Uniprot (https://www.uniprot.org/uniprot/). Positive and negative values correspond to genes up- and down-regulated. The treatments in brackets were performed as controls.

**Table S3** Lipid species related to phospholipids metabolism based on the top 10 VIP (variable importance on projection) scores from partial least squares discriminant analysis (PLS-DA) (*p* -value < 0.001).

| **Treatments** | **Lipids species** | **VIP** | **Log_2_FC** | **Up or Down** |
| --- | --- | --- | --- | --- |
| **-K+I vs (-K+M)** | PE(C14:0, C18:1) | 1.46 | -4.06 | Down |
|  | PC(C18:4, C24:1) | 1.40 | -2.94 | Down |
|  | PA(C22:5, C24:1) | 1.39 | -0.64 | Down |
|  | PC(C20:2, C18:1) | 1.36 | -3.47 | Down |
|  | PS(C20:2, C24:1) | 1.35 | -1.43 | Down |
|  | PC(C16:0, C24:0) | 1.34 | -2.47 | Down |
|  | PC(C22:4, C22:5) | 1.31 | -0.89 | Down |
|  | PE(C22:0, C18:0) | 1.28 | -1.84 | Down |
|  | PE(C22:4, C24:0) | 1.27 | -0.76 | Down |
|  | PS(C18:0, C24:0) | 1.25 | -1.00 | Down |
| **+K+I vs (+K+M)** | PC(C22:4, C22:5) | 1.78 | -0.71 | Down |
|  | PG(C16:0, C0:0) | 1.64 | 1.04 | Up |
|  | PC(C22:2, C22:6) | 1.61 | -0.94 | Down |
|  | PC(C22:5, C22:6) | 1.59 | 0.98 | Up |
|  | PC(C20:1, C18:1) | 1.47 | -0.92 | Down |
|  | PA(C10:0, C13:0) | 1.36 | -0.60 | Down |
|  | PA(C22:5, C24:1) | 1.34 | -0.40 | Down |
|  | PE(C22:5, C24:1) | 1.28 | -0.60 | Down |
|  | PC(C18:0, C24:0) | 1.27 | -1.29 | Down |
|  | PC(C18:2, C24:1) | 1.22 | -0.92 | Down |
| **-K+I vs (+K+I)** | PC(C22:5, C22:6) | 1.59 | -3.84 | Down |
|  | PE(C14:0, C15:0) | 1.57 | -5.06 | Down |
|  | PS(C20:2, C24:1) | 1.55 | -1.69 | Down |
|  | PC(C22:4, C22:5) | 1.52 | -0.97 | Down |
|  | PC(C18:4, C24:1) | 1.45 | -2.64 | Down |
|  | PS(C20:3, C24:1) | 1.41 | -0.51 | Down |
|  | PA(C22:4, C24:0) | 1.40 | -1.18 | Down |
|  | PC(C22:2, C22:6) | 1.35 | -2.47 | Down |
|  | PC(C20:3, C24:1) | 1.33 | -3.84 | Down |
|  | PE(C22:0, C18:0) | 1.32 | -1.43 | Down |

**Note:** The treatments in brackets is performed as control; PE: phosphatidylethanolamine; PC: phosphatidylcholine; PA: phosphatidic acid; PS: phosphatidylserine; PG: phosphatidylglycerol. Different numbers of carbon atoms represent the different lipids species.

**Table S4** Morphological parameters of chloroplasts (measured at 5 days after *S. oryzae* inoculation).

| **Treatments** | | **Length**  **(μm)** | **Width**  **(μm)** | **Area**  **(μm^2^)** | **L/W** |
| --- | --- | --- | --- | --- | --- |
| **+M** | **-K** | 4.87 a | 1.50 c | 7.41 c | 3.34 b |
|  | **+K** | 5.31 a | 1.36 c | 6.93 c | 3.91 a |
| **+I** | **-K** | 5.27 a | 3.33 a | 14.83 a | 1.58 d |
|  | **+K** | 4.62 a | 2.80 b | 11.82 b | 1.66 c |
| ***ANOVA*** | |  |  |  |  |
| **Inoculation(I)** | | ns | ** | ** | ** |
| **Treatments(K)** | | ns | * | ns | ns |
| **I×K** | | ns | ns | ns | ns |

**Note:** +M, +I denote mock inoculation (with sterile water) and *S. oryzae* inoculation, respectively. L/W, length ratio to width; **, * stand for *P*< 0.01 and *P*< 0.05 respectively, ns represents no significant difference, same as below (Duncan’s multiple range test (*P* < 0.05)).

**Table S5** Photosynthetic characteristics of flag leaf.

| **Treatments** | | ***A***  ***(μmol m^-2^ s^-1^)*** | ***J*_max_**  **(μmol m^-2^ s^-1^)** | ***V*_c,max_**  **(μmol m^-2^ s^-1^)** | **φPSII** | **F_v_/F_m_** |
| --- | --- | --- | --- | --- | --- | --- |
| **+M** | **-K** | 15.4 b | 138.7 b | 84.6 b | 0.17 ab | 0.81 ab |
|  | **+K** | 20.6 a | 188.2 a | 101.7 a | 0.24 a | 0.82 a |
| **+I** | **-K** | 12.0 c | 129.3 b | 61.7 c | 0.13 b | 0.78 b |
|  | **+K** | 16.5 b | 177.9 a | 88.0 b | 0.20 a | 0.82 a |
| ***ANOVA*** | |  |  |  | * |  |
| **Inoculation(I)** | | ** | ns | ** | ** | * |
| **Treatments(K)** | | ** | ** | ** | ns | ns |
| **I×K** | | ns | ns | ns | * | ns |

**Note:** The photosynthetic characteristics were analyzed for four tagged leaves in each treatment in the morning (9:00–11:30) at 5 days upon *S. oryzae* inoculation. A, the net photosynthetic rate; J_max_; maximum electron transport rate, V_cmax_; maximum carboxylation rate, φPSII; the effective quantum efficiency of PSII; F_v_/F_m_; Maximum quantum efficiency of PSII photochemistry. **, * stand for *p* < 0.01 and *p* < 0.05 respectively, ns represents no significant difference, same as below (Duncan’s multiple range test (*p* < 0.05)).

**Reference**

Bai S, Liu J, Chang C, Zhang L, Maekawa T, Wang Q, Xiao W, Liu Y, Chai J, Takken FLW, Schulze-Lefert P and Shen Q. Structure-Function Analysis of Barley NLR Immune Receptor MLA10 Reveals Its Cell Compartment Specific Activity in Cell Death and Disease Resistance. *PLOS Pathogens*. 8 (2012) e1002752.
